# EVI1 dysregulation: impact on biology and therapy of myeloid malignancies

**DOI:** 10.1038/s41408-021-00457-9

**Published:** 2021-03-22

**Authors:** Christine Birdwell, Warren Fiskus, Tapan M. Kadia, Courtney D. DiNardo, Christopher P. Mill, Kapil N. Bhalla

**Affiliations:** Division of Cancer Medicine, Department of Leukemia, The University of Texas M. D. Anderson Cancer Center, Houston, TX 77030 USA

**Keywords:** Acute myeloid leukaemia, Acute myeloid leukaemia

## Abstract

Ecotropic viral integration site 1 (*Evi1*) was discovered in 1988 as a common site of ecotropic viral integration resulting in myeloid malignancies in mice. EVI1 is an oncogenic zinc-finger transcription factor whose overexpression contributes to disease progression and an aggressive phenotype, correlating with poor clinical outcome in myeloid malignancies. Despite progress in understanding the biology of EVI1 dysregulation, significant improvements in therapeutic outcome remain elusive. Here, we highlight advances in understanding EVI1 biology and discuss how this new knowledge informs development of novel therapeutic interventions. EVI1 is overexpression is correlated with poor outcome in some epithelial cancers. However, the focus of this review is the genetic lesions, biology, and current therapeutics of myeloid malignancies overexpressing EVI1.

## MECOM locus discovery

**Fig. 1:**
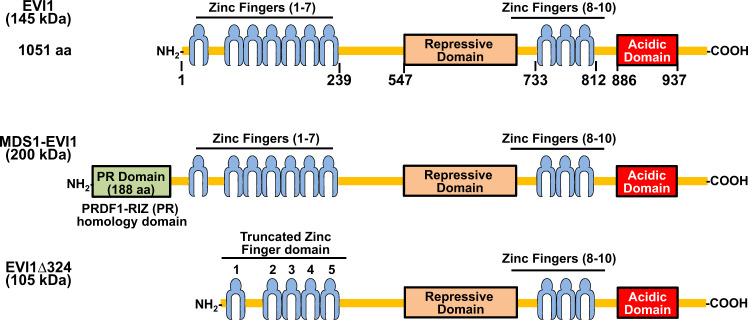
Schematic of the MDS and EVI1 (MECOM) locus proteins. C2H2-zinc finger motifs are shown in blue. Zinc finger motifs 1–7 and motifs 8–10 form the N-terminal and C-terminal zinc finger domains respectively. The repressive domain and acidic domain are depicted in tan and red, respectively. The numbers beneath the schematic indicate the amino acid positions of the zinc finger domains, the repressive domain and the acidic domain of EVI1.

*Evi1* was discovered by Mucenski et al. as a common site of ecotropic viral integration in mice that caused virally induced myeloid malignancies^1^. Through its rearrangements in human acute myeloid leukemia (AML), the human *EVI1* gene was mapped to the long arm of chromosome 3 at q 26.2 (3q26.2)^2^. The *EVI1* gene in humans is ~92% homologous to the mouse *Evi1*^2^. EVI1 is encoded from the MDS1 and ecotropic viral integration site 1 (EVI1) complex locus (MECOM), which includes several alternative transcripts^3^. *EVI1* exists either as a shorter single gene or as spliced to the short myelodysplastic syndrome 1 (*MDS1*) gene, present more than 350 kb upstream to *EVI1*, creating the longer *MDS1-EVI1* gene^3^. The shorter isoform of *EVI1* is abundant and oncogenic^4,5^. A truncated variant of the *EVI1* transcript conserved in both mice and humans, *EVI1∆324*, lacks part of the first zinc finger domain and the ability to transform (Fig. 1)^4^.

### EVI1: domain-structure and function

Human EVI1 is a 145 kilo Dalton (kDa) protein that contains 1051 amino acids. EVI1 localizes to the nucleus and binds DNA through its zinc finger (ZF) domains^5^. EVI1 contains ten zinc fingers that are arranged in two separate sets, one N-terminal containing seven zinc fingers, another C-terminal containing three zinc fingers^5^ (Fig. 1). Through electrophoretic mobility shift assays and chromatin immunoprecipitation (ChIP) assays, the N-terminal ZF domain was determined to bind TAGA/TCTA or CAGAGA/TCTCTG GATA-like simple sequence repeats (SSRs)^5,6^. The C-terminal ZF domain recognizes a CCATATAA ETS-like motif^5,6^. In the region between the ZF domains, is the repressor region that contains the interaction sites for the co-repressor CtBP (C-terminal binding protein 1)^7,8^. EVI1 also contains an acidic domain at its C terminus^5^ (Fig. 1).

Disruption of the full-length *Evi1* transcript by mutagenesis in mice led to severe developmental defects in the heart and central nervous system, and homozygous mutants died at approximately embryonic day 10.5^9^. Additionally, adult mice with conditional knockout of *Evi1* had a marked reduction in their long-term hematopoietic stem cells (LT-HSCs), and upon transfer into irradiated mice were unable to engraft and repopulate efficiently^10^. The self-renewal ability of LT-HSCs is linked to EVI1 expression, and many LT-HSC-associated genes have EVI1 binding sites in their regulatory regions^11^. Furthermore, increased EVI1 expression is a common immortalizing factor of murine primary bone marrow after retroviral infection^12^. For example, MSCV integration promoted increased expression of EVI1 causing immortalization of immature myeloid cells, but they were unable to induce leukemia in transplanted hosts^12^. Thus EVI1 supports HSC self-renewal, but EVI1 expression alone is not enough to drive leukemogenesis^12,13^.

In addition to LT-HSC self-renewal, expression of EVI1 blocks hematopoietic differentiation of the granulocyte, erythroid, dendritic, and monocytic lineages^14,15^. EVI1 expression in primary mouse myeloid progenitor cells upregulated HSC-associated genes and decreased DNA replication and repair genes^14^. *EVI1* transcripts are decreased in human CD34^+^ cells after stimulation of differentiation induced by cytokine administration, suggesting that downregulation of EVI1 is an important step in terminal differentiation of many hematopoietic lineages^15^. Forced expression of *Evi1* in the mouse bone marrow cell line 32Dcl3 inhibits differentiation response to granulocytes and erythrocytes due to granulocyte colony-stimulating factor (G-CSF) and erythropoietin, respectively^16,17^. Extrinsic EVI1 expression blocked G-CSF-induced differentiation through transcriptional repression of the lineage-specific gene myeloperoxidase and the myeloid transcription factors C/EBPα (CCAAT enhancer binding protein alpha) and RUNX1 (runt-related transcription factor 1, also known as AML1)^16,18^. Erythroid differentiation was blocked by EVI1 through binding and subsequent inhibition of transcriptional activity of the myeloid transcription factors GATA1 (GATA binding protein 1) and PU.1 (transcription factor PU.1)^17,19^. In the megakaryocyte lineage, EVI1 is expressed in early precursor cells^20^. In a transgenic mouse model recapitulating human inv^3^(q21q26) AML that overexpresses EVI1 and also has GATA2 haploinsufficiency, EVI1 and GATA2 dysregulation together skewed hematopoiesis toward the megakaryocyte lineage more so than EVI1 overexpression alone^21^. This suggests that EVI1 may work in concert with other factors to promote the megakaryocyte lineage^21^. In general, for most myeloid lineages, EVI1 functions to promote a stem or early progenitor transcriptional program^11,14^. Forced EVI1 expression maintains the stem-like program while simultaneously suppressing myeloid transcription factors involved in myeloid differentiation^16,18,19,21^. Notably, endogenous EVI1 is generally downregulated under normal differentiation^14,15^. However, the degree to which endogenous EVI1 blocks differentiation and what factors normally downregulate EVI1 during differentiation largely remain an unknown.

### MDS1-EVI1 and EVI1Δ324

In 1994, Nucifora et al. identified a transcript of unknown function that they termed *MDS1*, which formed a fusion protein with RUNX1 and/or EVI1 in several myelodysplastic syndrome (MDS) patients^22^. Currently, the function of the MDS1 protein itself is still unknown. In *MDS1-EVI1*, exon 2 of *MDS1* is fused in-frame to *EVI1* exon 2, which adds 188 amino acids upstream of the normal start codon of *EVI1* in exon 3^22^. A part of these extra N-terminal amino acids contains the PR domain, which shares homology with the B cell factor positive regulatory domain 1-binding factor (PRD1-BF1) and retinoblastoma binding protein RIZ1^3,12^. The PR domain is related to a subset of the methyltransferase SET domains^3,23^ (Fig. 1). The combination of a PR domain and zinc finger domains in MDS1-EVI1 makes it a part of the PRDM (PR/SET domain) family and thus is also called PRDM3, which was characterized as a mono-methyl H3K9 methyltransferase^23^. Although the specific role of MDS1-EVI1 is not always separated from the role of EVI1, loss of MDS1-EVI1 is also associated with embryonic lethality, developmental defects, and dysregulation of hematopoiesis^10,15^.

*EVI1Δ324* is a variant transcript of *EVI1* with an internal 972 nucleotide deletion that removes the 6th and 7th zinc finger from the N-terminal ZF domain^4^ (Fig. 1). ChIP assays with FLAG-tagged EVI1 or EVI1Δ324 in an ovarian carcinoma cell line (SKOV3) showed an ~71% overlap in binding peaks between the two^24^. Additionally, the transcriptional profile of HeLa cells overexpressing EVI1-FLAG or EVI1Δ324-FLAG was almost identical^24^. However, EVI1Δ324 does not replicate the transformative effects of EVI1 in rat fibroblasts, and is not known to have oncogenic activity nor is it linked currently with any myeloid malignancy^24^.

## *EVI1* regulation

### Epigenetic regulation of EVI1

The region 5′ of *EVI1* contains two CpG islands, one close to the transcription start site of *EVI1* and a second located near *MDS1*^25^. In an AML cell line that has low EVI1 expression, the CpG islands related to *EVI1* and *MDS1* had a marked increase in methylation, suggesting that *EVI1* expression can be regulated by methylation in AML cells^25^. Furthermore, AML cell lines with high EVI1 expression displayed active chromatin marks, with histone acetylation and enrichment of H3K4me3 (histone 3 lysine 4 tri-methylation) at the *EVI1* promoter. In contrast cell lines with low EVI1 expression have enrichment of the repressive histone mark H3K27me3 (histone 3 lysine 27 tri-methylation)^25^.

### EVI1 promoter

**Fig. 2:**
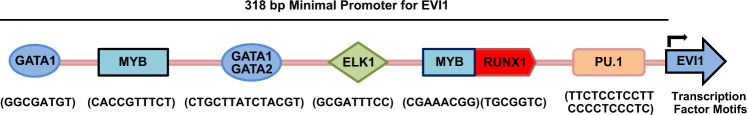
Schematic representation of the 318-bp minimal promoter of EVI1 in humans. Within the upstream EVI1 promoter, the positions and nucleotide sequences/binding sites of specific transcription factors are shown.

The minimal promoter of *EVI1* was localized to a 318 nucleotide region 5′ of the *EVI1* transcription start site that does not contain a traditional TATA or CAAT box^26^ (Fig. 2). In the *EVI1* minimal promoter, analysis of binding motifs and site directed mutagenesis identified active binding motifs for RUNX1, ELK1 (ETS transcription factor ELK1), RELA (RELA proto-oncogene, NF-κB Subunit), GATA1, and MYB (MYB proto-oncogene, transcription factor). Knockdown of RUNX1 and/or ELK1 in HEL cells decreased *EVI1* mRNA and protein levels^26^. Furthermore, interactions between RUNX1 and EVI1 at the minimal promoter appear to positively regulate EVI1 activity^26^. MDS1-EVI1 and EVI1Δ324 bind further downstream of the minimal promoter of *EVI1* and reduce its transcription^26,27^. MDS1-EVI1 and EVI1∆324 are reported to be co-expressed with EVI1^15,24^. Although not further studied, present upstream of the minimal *EVI1* promoter are the consensus binding motifs for GATA1, GATA2, and C/EBPα, suggesting that MDS1-EVI1 and EVI1∆324 may work in concert with other transcription factors to repress EVI1^27^. CML (chronic myeloid leukemia) blast crisis patient-derived cells express high EVI1 and β-catenin levels^28^. Knockdown of β-catenin or its related co-transcription factor LEF1 (lymphoid enhancer factor 1) decreased EVI1 levels^28^. Bioinformatic analysis indicated two potential tandem LEF1/β-catenin-binding sites present 1.44 kb upstream of *EVI1*, which are bound by LEF1, as determined by ChIP assays^28^. Additional studies are needed to further clarify regulation of *EVI1* by LEF1/β-catenin, RUNX1, GATA1, and/or ELK1.

### Post-translational modifications on EVI1

EVI1 has been reported to be phosphorylated at serine 196 (S196), S538, S858, and S860^29,30^. Stable isotope labeling of amino acids followed by mass spectrometry (SILAC-MS) identified EVI1-associated proteins. CK2 (casein kinase 2) was confirmed to phosphorylate EVI1 residues S538 and S858. Loss of phosphorylation was mediated by PP1α (protein phosphatase 1 alpha), and it decreased DNA-binding by the C-terminal ZF domain^29^. In contrast, phosphorylation of S196 on the 6th zinc finger in the N-terminal ZF domain decreases DNA binding and repression by EVI1 of promoters containing GATA-like motifs^30^. Although phosphorylation of Ser858 and Ser860 did not affect EVI1 DNA binding, loss of these phosphorylations blunted EVI1 transcriptional repression after cellular stress through reduced interaction of EVI1 with co-repressor CtBP1^31^. EVI1 is also acetylated by CBP (CREB binding protein or KAT3A)/p300 (EP300, or KAT3B) and PCAF (P300/CBP-associated factor or KAT2B)^32^. CBP-induced acetylation increased EVI1 transcriptional activity in luciferase assays^32^. In contrast, PCAF-mediated acetylation of EVI1 has been reported to exhibit opposing effects on EVI1 activity. Co-expression of EVI1 with PCAF abrogated EVI1-mediated Bcl-xL expression, suggesting that EVI1 acetylation blocked EVI1 transactivation activity at the Bcl-xL promoter^33^. In contrast, PCAF-mediated acetylation of K564 on EVI1 increased its ability to transactivate GATA2, and this ability was lost in a K564A mutant that cannot be acetylated^34^. Overall, it is unclear whether these post-translational modifications can occur simultaneously, or whether one modification can hinder the acquisition of another.

## Transcriptional regulation by EVI1

### Transcriptional repression by EVI1

EVI1 co-immunoprecipitates with the H3K9me3 methyltransferase SUV39H1 (suppressor of variegation 3–9 homolog 1) and the related H3K9me1/2 methyltransferase G9a (euchromatic histone lysine methyltransferase 2)^35,36^ (Table 1A). EVI1 and SUV39H1 interaction required the N-terminal ZF domain of EVI1 and the C-terminal domain of SUV39H1. Histone methyltransferase assays showed SUV39H1 had methyltransferase activity alone or in a complex with EVI1. Furthermore, it was observed by the Nucifora and Delwel groups that EVI1-mediated repression of a GAL4 luciferase construct was enhanced by SUV39H1 co-expression^35,36^.

**Table 1:** (A) EVI1 interactions with epigenetic regulators. (B) Biology of direct interaction of EVI1 with other transcription factors.

(A)		EVI1 interaction domain	Cellular models studied	Ref
DNA methyltransferase				
DNMT3A		N-terminal zinc finger domain	293T, SB1690CB	^38,39^
DNMT3B		N-terminal zinc finger domain	293T, SB1690CB	^38,39^
Histone methyltransferase				
SUV39H1		N-terminal zinc finger domain	φE, 293T, HeLa	^35,36^
G9a		N-terminal zinc finger domain	φE, 293T, HeLa	^35,36^
EZH2		N-terminal zinc finger domain	THP-1, Jurkat, AML samples	^37^
Histone acetyltransferase				
CBP		Central region	Cos7	^32^
PCAF		N-terminal region/C-terminal region	Cos7, HT-29, UCSD-AML1	^32–34^

(B)	TFs	Activity	EVI1 interaction domain	Cellular models studied	Biological outcome	Ref
	Myeloid					
	RUNX1	Down	8th zinc finger and central domain	NIH-3T3, 32Dcl3, 293T, K562	Blocks myeloid differentiation	^43^
	GATA1	Down	1st and 6th zinc fingers	32DEpo1, 32Dcl3, Cos7, AML14.3D10	Blocks myeloid differentiation	^42^
	PU.1	Down	6th and 7th zinc fingers	32Dcl3 and 293T	Blocks myeloid differentiation	^19^
	General					
	SMAD3	Down	1st–7th zinc fingers	32Dcl3	Blocks TGF-β responsiveness	^41^

(A) Epigenetic regulator proteins experimentally determined to interact with EVI1.

*EVI1* ecotropic viral integration site 1, *N-ter ZF domain* N-terminal zinc finger domain, *DNMT3A/B* DNA methyltransferase 3A/B, *SUV39H1* suppressor of variegation 3-9 homolog 1, *G9a* euchromatic histone lysine methyltransferase 2, *EZH2* enhancer of zeste 2, *CBP* CREB binding protein a.k.a. KAT3A, *PCAF* P300/CBP-associated factor a.k.a. KAT2B.

(B) Transcription factors experimentally determined to directly interact with EVI1, the interacting domain of EVI1 involved and the implications of the interaction on the activity of the transcription factor.

*TFs* transcription factors, *EVI1* ecotropic viral integration site 1, *RUNX1* RUNX family transcription factor 1, *GATA1* GATA binding protein 1, *PU.1* transcription factor PU.1, *SMAD3* SMAD family member 3, *NF-κB p65* nuclear factor kappa B family member p65.

EVI1 represses PTEN (phosphatase and tensin homolog) through its N-terminal ZF domain and via recruitment of the polycomb repressor complex 2 (PRC2), including EZH2 (enhancer of zeste 2), by binding upstream of the PTEN transcription start site^37^. This increased accumulation of the repressive H3K27me3 mark and reduced histone acetylation at the PTEN locus has been observed in human AML patient samples^37^.

EVI1 interacts through its N-terminal ZF domain with the de novo DNA methyltransferases DNMT3A and 3B^38,39^. EVI1 expression correlated with differential hypermethylation of over 200 genes, as compared to normal CD34^+^ cells, or to a previously reported DNA methylation profile in a separate cohort of 344 AML patients^39^. Unbiased motif analysis of differentially methylated gene promoters showed an enrichment of the motif recognized by the N-terminal ZF domain of EVI1^39^. DNMT3A was also found to be highly expressed in EVI1-high AML samples compared to other AML subtypes. EVI1 expression levels correlated positively with a stronger hypermethylation signature in AML patient samples^39^.

### Interaction with co-repressor CtBP

A region just left to the C-terminal ZF domain of EVI1 was associated with transcriptional repression activity of EVI1 and shown to be required for EVI1 transformation of rat fibroblasts^40^. This region was also critical for EVI1 repression of TGF-β (transforming growth factor beta) signaling and was thus termed the repressive domain (Rp) (Fig. 1)^41^. Two consensus binding motifs for the transcriptional co-repressor CtBP were identified in the EVI1 Rp region. The PLDLS sequence at the residue 584 of EVI1 is the major site of CtBP interaction^7,8^. Mutation of the CtBP binding site at residue 584 abolished the ability of EVI1 to repress TGF-β-mediated growth arrest and transformation of rat fibroblasts^7,8^.

### Repression of other transcription factors by EVI1

EVI1 can also directly bind several transcription factors and inhibit their activity (Table 1B). EVI1 was able to repress GATA1-mediated activation of a synthetic promoter. However, EVI1 does not bind to the canonical GATA1 motif^42^. Instead, EVI1 zinc fingers one and six directly interact with the C-terminal zinc finger of GATA1 in GST-fusion pull-down assays. Also, EVI1 interaction with GATA1 decreased GATA1 DNA-binding ability. Mutation of EVI1 zinc fingers one and six abolished GATA1 interaction and restored differentiation potential to 32Dcl3 cells in response to erythropoietin^42^.

The 6th and 7th zinc finger of EVI1 was shown to directly interact with the C-terminal ETS-domain of PU.1 through co-immunoprecipitation and GST-fusion pull-down assays. Binding of EVI1 to PU.1 did not prevent DNA-binding ability of PU.1; instead it blocked association of PU.1 with c-Jun (Jun Proto-Oncogene), a subunit of the transcription factor AP-1. Mutation of the 6th and 7th EVI1 zinc fingers mitigated EVI1 interaction with PU.1 and restored differentiation potential to 32Dcl3 cells in response to G-CSF^19^. The 8th zinc finger in the C-terminal ZF domain of EVI1 was shown to interact with RUNX1^43^. Binding of EVI1 repressed transcriptional activity of RUNX1 by decreasing its DNA-binding^43^. However, RUNX1 interaction with EVI1 had no effect on EVI1 DNA-binding. EVI1 interacts with the transcription factor SMAD3 through its N-terminal ZF domain^41^. EVI1 interaction repressed SMAD3 activity leading to blocked TGF-β mediated growth inhibition^41^.

### Transcriptional activation by EVI1

A number of gene targets are upregulated by EVI1 (Table 2). EVI1 interaction with histone acetyltransferases has been reported to promote EVI1-mediated transcriptional activation^32,34^. EVI1 interaction with AP-1 subunits c-Fos and c-Jun was noticed as early as 1994 by Tanaka et al.^44^. EVI1-expressing cells exhibited increased c-Fos and c-Jun levels, and the C-terminal ZF domain of EVI1 was critical for activation of the c-Fos promoter^44^. Loss of EVI1 decreased c-Fos occupancy on the DNA, suggesting that EVI1 and AP-1 may act cooperatively at some loci^6^. A SILAC-MS screen also confirmed c-Fos and c-Jun interaction with EVI1^29^. This screen also identified several additional transcription factors and co-factors that interact with EVI1, and 65% of EVI1-regulated genes were upregulated^6,29^. This highlighted the role of EVI1 as a transcriptional activator.

**Table 2:** Transcriptional targets of EVI1.

Gene	Activity/levels	Regulation	Cellular models studied	Biological outcome	Ref
MYC	Up	Transcriptional upregulation	SKOV3, HeLa	Active metabolism and apoptosis resistance	^73^
BcL-xL	Up	Transcriptional upregulation	HT-29, 293T AML samples	Apoptosis resistance	^33^
GPR56	Up	Transcriptional upregulation	UCSD-AML1, HNT-34 AML samples	Apoptosis resistance	^71^
ITGA6	Up	Transcriptional upregulation	UCSD-AML1, HNT-34 AML samples	Apoptosis resistance	^72^
c-Fos	Up	Transcriptional upregulation	P19, SKOV3, HeLa	Activates AP-1	^44^
PBX1	Up	Transcriptional upregulation	HEL Primary murine BM	Maintains AML stem cell phenotype	^68^
GATA2	Up	Transcriptional upregulation	EML-C1, HEL Primary mouse EC	Maintains AML stem cell phenotype	^34^
C/EBPα	Down	Transcriptional repression	32Dcl3, EML, DA-1, U937	Blocks differentiation	^17,18^
RUNX1	Down	Transcriptional repression	32Dcl3	Blocks differentiation	^16^
PTEN	Down	Transcriptional repression	Primary murine BM AML samples	Activates metabolism and apoptosis resistance by PI3K/AKT/mTOR pathway	^37^

Transcriptional targets of EVI1, the effect on the activity/levels of each target, and the biologic consequence of EVI1-mediated transcriptional regulation on the target genes.

*EVI1* ecotropic viral integration site 1, *BM* bone marrow, *EC* embryonic cells, *PI3K* phosphoinositide 3-kinase, *AKT* AKT serine/threonine kinase, *mTOR* mechanistic target of rapamycin kinase, *PTEN* phosphatase and tensin homolog, *MYC* MYC proto-oncogene, *BHLH transcription factor, BcL-xL* BCL2 Like 1, *GPR56* adhesion G protein-coupled receptor G1, *ITGA6* integrin subunit alpha 6, *c-Fos* Fos proto-oncogene, AP-1 transcription factor subunit, *PBX1* PBX homeobox 1, *GATA2* GATA binding protein 2, *C/EBP* CCAAT enhancer binding protein, *CDK2* cyclin dependent kinase 2.

## EVI1 dysregulation in myeloid leukemia

### Chromosome 3 lesions leading to EVI1 overexpression

**Fig. 3:**
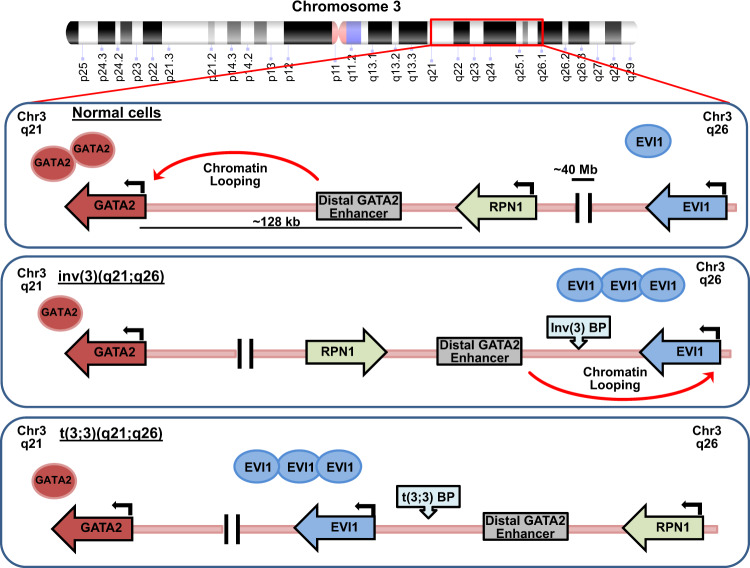
Schematic of the q21-26 locus on chromosome 3 in normal cells and cells with inv(3)(q21q26) or t(3;3)(q21q26). In both inv(3)(q21q26) or t(3;3)(q21q26), the breakpoints lead to juxtaposition of a region surrounding the distal GATA2 enhancer and the RPN1 gene in 3q21 with the EVI1 gene in 3q26. Breakpoints occur 3′ of the EVI1 gene in the inv(3)(q21q26) setting, whereas they occur 5′ of the EVI1 gene in the case of t(3;3)(q21q26). In both types of 3q21q26 rearrangement, the GATA2 enhancer induces EVI1 gene transcription instead of GATA2 expression and thus promotes leukemogenesis.

In the World Health Organization (WHO) classification of AML and related neoplasms, inversion or translocation of chromosome 3 at the MECOM locus [inv3(3;3)(q21q26)/inv,(3) t(3;3)(q21;q26.2)/t(3;3)] have been recognized as recurrent genetic abnormalities^45^ (Fig. 3). Inv(3)/t(3;3) is observed in ~1–2.5% of MDS and in a similar percentage of AML patients^46,47^. Inv(3)/t(3;3) rearrangements can also be observed in up to 25–40% of CML patients in blast crisis^48,49^. Despite their existence as distinct clinical entities, MDS, AML, and CML with inv(3)/t(3;3) rearrangements have similar cytogenetic abnormalities, molecular alterations, pathological features, and poor prognosis^46,50–52^. In inv,(3) breaks most frequently occur in a region between *RPN1* (Ribophorin 1) and *C3orf27*, downstream of *GATA2*, that contains a distal GATA2 hematopoietic enhancer (−77 kb, G2DHE) and the region between *C3orf50* and the first exon of the MECOM locus that encodes for the *MDS1-EVI1* transcript (Fig. 3)^53^. The *EVI1* and *EVI1Δ324* transcripts remain intact, but the *MDS1-EVI1* transcript is frequently not expressed^53^. In t(3;3), the breakpoint frequently occurs in between the *MDS1* promoter and the first *EVI1* exon, and *MDS1-EVI1* transcript is frequently lost (Fig. 3)^46^. In 2014, the Delwel group and the Yamamoto group identified that a new super enhancer of ~40 kb is formed from repositioning of the GATA2 distal hematopoietic enhancer that drives increased *EVI1* expression in inv(3)/t(3;3) AML^53,54^. The new enhancer region generated by the chromosomal rearrangement was noted to contain a 9-kb region with a p300 binding site that interacts with the *EVI1* promoter, and removal of this binding site attenuates *EVI1* expression^53^. Transgenic mice in which the human inv(3) chromosomal abnormality was recapitulated through a bacterial artificial chromosome developed leukemia, but if the relocated GATA2 enhancer region was deleted, *EVI1* expression declined and leukemia did not develop^54^. These seminal studies confirmed that *EVI1* dysregulation in response to chromosomal rearrangements in myeloid disease occurred not from the typical generation of a fusion transcript, but rather from “enhancer hijacking”^53,54^. This highlighted a two-fold impact, one leading to overexpression of EVI1 and the second causing haploinsufficiency of GATA2, as it is no longer expressed in the rearranged chromosome^53^.

### Atypical 3q26 rearrangements

In ~0.5–1% of AML and MDS patients, atypical chromosome 3 rearrangements occur involving the MECOM locus^55^. Most atypical 3q26 rearrangements have levels of *EVI1* overexpression comparable to inv(3)/t(3;3) cases, similar phenotypic changes, and share a poor prognosis^46,55^. Atypical 3q26 rearrangements include, but are not limited to t(2;3)(q21;q26), t(3;7)(q26;q24), t(3;8)(q26;q24), and t(3;6)(q26;q25), which involve THADA (THADA armadillo repeat containing), CDK6 (cyclin dependent kinase 6), MYC (V-Myc avian myelocytomatosis viral oncogene homolog), and ARID1B (AT-rich interaction domain 1B), respectively^55^. Similar to the “enhancer hijacking” of the GATA2 distal hematopoietic enhancer in inv(3)/t(3;3), atypical 3q26 rearrangements also seem to overexpress *EVI1* through repurposing of enhancer elements from the translocation partners. In ten cases of atypical 3q26 rearrangements, *EVI1* was overexpressed and the translocation partner whose enhancer was repurposed had decreased expression, with the exception of *MYC* in t(3;8)(q26;q24)^55^.

### EVI1 fusion proteins

**Fig. 4:**
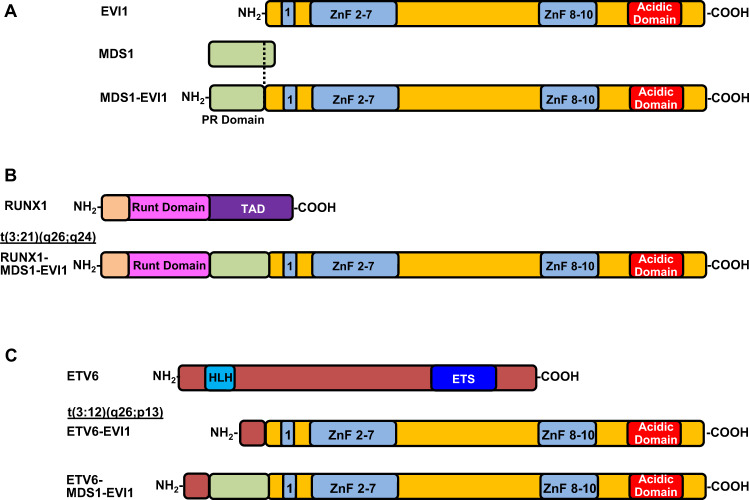
EVI1 and EVI1 fusion proteins. Schematic diagram of the EVI1, MDS1, MDS1-EVI1, RUNX1, RUNX1-MDS1-EVI1, ETV6, ETV6-EVI1, and ETV6-MDS1-EVI1 proteins.

Several translocations involving the MECOM locus do result in the generation of fusion proteins. The two most common being t(3;12)(q26;p13) and t(3;21)(q26;q24) that result in ETS variant transcription factor 6 (also TEL)-EVI1 and RUNX1-MDS1-EVI1 fusion proteins, respectively^22,56^ (Fig. 4). Both translocations are rare, found in less than 1% of myeloid malignancies^56,57^. Fluorescent in situ hybridization demonstrated that the t(3;12) breakpoints are between the *ETV6* exon 2 and 3 and on heterogeneous regions in 3q26, both 3′ and 5′ of *MDS1* as well as in between *MDS1* and *EVI1*^56^. The resulting translocation fuses the first two exons of *ETV6* with the entire *MDS1-EVI1* or *EVI1* transcript. Since no known functional domain of ETV6 is added to EVI1 in the fusion protein, it is thought that the oncogenic properties of the fusion protein come from the inappropriate expression and function of EVI1 driven by the ETV6 promoter^56^. In line with this, similar to other 3q26 rearrangements, myeloid malignancies expressing the ETV6-EVI1 fusion are associated with dysmegakaryopoiesis and poor prognosis^58^. The t(3;21) can generate RUNX1-MDS1-EVI1 fusion protein, where the DNA-binding RUNT domain of RUNX1 is fused to the whole MDS1-EVI1 protein (Fig. 4)^59^. Expression of the RUNX1-MDS1-EVI1 protein is associated with disruption of RUNX1 and EVI1 regulatory networks. This is thought to be partly achieved by transcriptional repression of RUNX1 targets through recruitment of co-repressors by EVI1 in the fusion protein^57^. In mouse models of conditional RUNX1-MDS1-EVI1 expression or transplant models, the fusion protein is associated with development of hematopoietic dysplasia and acute megakaryoblastic leukemia^60^.

### EVI1 overexpression without chromosome 3 aberrations

Aberrant EVI1 expression can also occur in the absence of chromosome 3 rearrangements. EVI1 overexpression is observed in ~8–10% of MDS, 8% of de novo AML, and 30% of advanced CML, but it is unclear here how EVI1 overexpression occurs^61^. Several ChIP studies have shown that mixed lineage leukemia (MLL) and MLL fusion proteins, including MLL-AF9 and MLL-ENL bind to the *EVI1* regulatory region, resulting in increased EVI1 expression^62,63^. In a recent report in which MLL-AF9 fusion gene was expressed either in murine Sca^−^Kit^+^ (LSK) HSCs or in granulocyte monocyte precursors (GMPs), LSK-MLL-AF9 cells had significantly higher levels of *Evi1* than GMP-MLL-AF9 cells^64^. Additionally, AMLs with high EVI1 expression have been shown to be associated with inferior relapse-free and overall survival^65^.

## Biologic consequences of 3q lesions and EVI1 overexpression

### Genomic instability

Utilizing SILAC-MS studies to determine EVI1 interaction partners, Bard-Chapeau et al. observed enrichment in protein domains associated with DNA repair, chromatin remodeling, and transcription^29^. Furthermore, the EVI1 N-terminal ZF domain binds to GATA-like SSRs, and EVI1 ChIP analysis revealed an increase in recombination rates near EVI1 bound SSR^13,24^. How EVI1 increases genomic instability is not well characterized beyond its protein interactions. However, a gene therapy study using a Maloney murine leukemia virus vector to express NADPH-oxidase conducted in two patients to treat chronic granulomatous disease unfortunately caused integration of the vector at the MECOM locus. The patients developed clonal expansion of myeloid cells bearing activating insertions in the MECOM locus and EVI1 overexpression. Both patients developed monosomy 7 in the dominate clone, suggesting that EVI1 could favor expansion of clones with monosomy 7 or that EVI1 could contribute to the genomic instability leading to monosomy 7^13^.

### Effect of EVI1 on hematopoietic stem cell proliferation/differentiation

EVI1 is known to directly interact with and repress the activity of a number of myeloid transcription factors including GATA1, PU.1, and RUNX1^17,19,42,43^. Enforced *Evi1* expression transcriptionally repressed C/EBP-α in murine hematopoietic cells^18^. EVI1-mediated repression of C/EBP-α was also observed in the murine hematopoietic progenitor cell line 32Dcl3^17^. Further confirmation that EVI1 represses C/EBP family members is needed through in vivo leukemia models and in patient-derived samples. EVI1 also regulates hematopoietic differentiation and proliferation through transcriptional repression of several miRNAs. EVI1 repressed miR-9 levels through binding to its regulatory region, recruiting DNMT3B, and inducing DNA methylation^66^. Decreased miR-9 led to increased levels of its target genes FOXO1 and 3 (Forkhead Box O1 and 3)^66^. EVI1 expression was also found to decrease miR-449A levels, and ChIP analysis showed EVI1 bound miR-449A regulatory region^67^. Repression of miR-449A by EVI1 increased expression of the miR-449A-targets Notch1 and Bcl-2 in human AML cell lines^67^.

EVI1 transcriptionally activates the hematopoietic proto-oncogene PBX1 (PBX homeobox 1) through binding to its promoter region^68^. Knockdown of PBX1 decreased EVI1-mediated transformation of primary mouse bone marrow cells^68^. Comparing tissues from wild type to those from EVI1^+/−^ and EVI1^−/−^ mice, at embryonic day 9.5, GATA2 expression was decreased in EVI1 depleted tissues^69^. EVI1 expression also correlated with high expression of megakaryocytic markers, including the thrombopoietin receptor MPL^70^. Furthermore, in a mouse model of EVI1 leukemia, thrombopoietin expression correlated with EVI1 expression, and double positive EVI1-thrombopoietin cells had enhanced secondary leukemia formation ability in a serial bone marrow transplant assay^70^. Collectively, in myeloid malignancies expressing EVI1, transcriptional alterations of specific myeloid transcription factors, and of miRNAs, contribute to myeloid dysplasia.

### Increased drug resistance

Several pathways have been implicated in EVI1-mediated resistance to apoptosis leading to drug-resistance. High EVI1 expression correlated with high expression of the anti-apoptotic Bcl-xL protein in CML patient samples^33^. Conversely, knockdown of EVI1 was shown to decrease Bcl-xL levels by approximately five fold^33^. EVI1 interactions with the microenvironment are also implicated in apoptosis-resistance. The adhesion molecules ITGA6 (integrin subunit alpha 6) and GPR56 (adhesion G protein-coupled receptor G1) are highly expressed in EVI1-positive AML, and their knockdown leads to increased apoptosis in response to Ara-C treatment and loss of RhoA (ras homolog family member A) signaling, respectively^71,72^. In AML, cells high EVI1 expression correlated with high MYC and BCL2 expression, with poorer clinical outcome^73^.

## Clinical phenotypes and outcome of EVI1-positive myeloid malignancies

MDS with EVI1 overexpression is commonly associated with dyserythropoiesis and with the presence of micro megakaryocytes^51^. Categorized as high risk, more than half of inv(3)/t(3;3) MDS patients with EVI1 overexpression progress to AML within ~2 years of diagnosis^46,47^. Furthermore, EVI1 overexpression correlates with shorter overall survival and poorer response to treatment^51^. Overall survival of patients with EVI1-positive MDS ranges from 13 to 17 months after diagnosis^46,47^. Like EVI1_-_positive MDS, AML with EVI1 overexpression often presents with myeloid dysplasia, particularly of the erythrocyte and megakaryocytic lineages^46,51^. Studies have also reported EVI1 expression as an independent prognostic factor for poorer overall survival in AML, and high EVI1 expression is associated with poorer response to therapy^46,74,75^. Several clinical studies have reported that, in 3q26-rearranged AML, the median overall survival after diagnosis remains approximately less than 1 year, whereas long-term overall survival is less than 15% (Table 3)^47,74,76^.

**Table 3:** Retrospective analysis of clinical outcome of patients with 3q26 genetic lesions.

Year	First author	*N*	CR (%)	Median OS (m)	1-year OS	Long term OS	Long term relapse probability	Ref
2010	Lugthart	79	31%	10.3	N.D.	5-year OS: 5.7%	5-year RFS: 4.3%	^46^
2010	Grimwade	69	36%	N.D.	N.D.	10-year OS: 3%	10-year CIR: 89%	^75^
2011	Sun	30	42%	8.9	33%	5-year OS: 3%	N.D.	^45^
2015	Wanquet	40	29%	10.6	N.D.	4-year OS: 3%	N.D.	^87^
2020	Sitges, M	61	29%	8.4	42%	4-year OS: 13%	N.D.	^76^

*N* number, *ORR* overall response rate, *CR* complete remission, *OS* overall survival, *m* months, *RFS* relapse-free survival, *CIR* cumulative incidence of relapse, *N.D.* not discussed.

EVI1-expressing CML may also be associated with megakaryocytic dysplasia^51^. EVI1 expression is rarely detected in the chronic phase of CML, but is readily detected in a significant proportion (25–40%) of blast crisis of CML, suggesting that acquisition of EVI1 expression can drive progression into blast crisis^48,49^. EVI1 expression in CML blast crisis is correlated with poor response to therapy, and has been linked with acquisition of resistance to tyrosine kinase inhibitors^48^.

### Monosomy 7 and MLL translocations

Loss of one copy of chromosome 7 (monosomy 7, −7) or deletion of the long arm of chromosome 7 (−7q) is observed in 30–70% of MDS and AML with inv(3)/t(3;3)^74^. Retrospective studies have shown that inv(3)/t(3;3) MDS/AML with −7/−7q display worse prognosis than inv(3)/t(3;3) alone^74,77^. Which genetic alteration occurs first is unclear, and likely varies on a case-by-case basis, given the heterogeneity of the myeloid malignancies. As noted above, in two cases where gene therapy activated EVI1 expression through retroviral insertion, both cases developed monosomy 7 in the dominant leukemic clone, suggesting that EVI1 at least favors events leading to monosomy 7^13^. The q arm of chromosome 7 contains several key genes whose haploinsufficiency is considered to be a loss of tumor-suppressor and thus contribute to leukemia transformation. These genes include EZH2 and MLL3, as well as the cytoplasmic cellular regulators SAMD9 (Sterile Alpha Motif Domain Containing 9) and SAMD9L^78^. Perhaps due to the ability of MLL and MLL-fusion proteins to upregulate EVI1 transcription, EVI1 overexpression can be observed in ~30% of cases with MLL translocation, and here EVI1 expression correlates with poor prognosis^63,65^.

### Transcription factor mutations

Approximately 20% of MDS and AML patients with inv(3)/t(3;3) express mutations in RUNX1^79^. Another transcription factor IKZF1 (IKAROS family zinc finger) is also mutated in up to 25% of cases of inv(3)/t(3;3) MDS or AML. Since IKZF1 is located on chromosome 7, IKZF1 mutations occur in clones without chromosome 7 deletions^77^. Although not a mutation, almost all MDS, AML, and CML with inv(3)/t(3;3) have GATA2 haploinsufficiency due to the re-location of the GATA2 distal hematopoietic enhancer^53,54^. This was shown to contribute to EVI1-driven leukemia transformation^21^. Of note, despite loss of expression from one allele of GATA2, 15% of inv(3)/t(3;3) can carry additional mutations in GATA2 on the non-rearranged allele^52,77^.

### Activating mutations in signaling pathways

A significant proportion of inv(3)/t(3;3) MDS and AML cases have activating mutations in RAS GTPase family member (NRAS or KRAS), or in other RAS-signaling pathway proteins, including PTPN11 (protein tyrosine phosphatase non-receptor type 11), and NF1 (neurofibromin 1), which promote dysregulated RAS signaling and uncontrolled proliferation^52,77,79^. These mutations are observed in 66–98% of inv(3)/t(3;3) MDS/AML^52,77,79^. A greater percentage of AML cases with inv(3)/t(3;3) AML carried RAS family mutations, as compared to the MDS cases^79^.

### Mutations in epigenetic machinery

Low frequency of mutations in DNMT3, TET2 (tet methylcytosine dioxygenase 2), and IDH1/2 (isocitrate dehydrogenase 1/2) were observed in AML or MDS with inv(3)/t(3;3)^46,51,77^. However, mutations in the polycomb group protein ASXL1 (ASXL transcriptional regulator 1) were reported in ~20% of AML cases with inv(3)/t(3;3)^77^. Mutations in splicing factors SF3B1 (splicing factor 3b subunit 1) and U2AF1 (U2 small nuclear RNA auxiliary factor 1) were also found in ~30–60% of inv(3)/t(3;3) MDS or AML cases^52,77^. The biologic impact of these ‘epimutations’ in myeloid malignancies on the transcriptional signature attributed to inv(3)/t(3;3) and EVI1 overexpression remains to be elucidated.

### Mutations inversely correlated with EVI1

Mutations in NPM1 (nucleophosmin 1) and C/EBP-α inversely correlate with inv(3)/t(3;3) and EVI1 expression^55,77,79^. Why EVI1 overexpression is not seen with NPM1 or C/EBP-α mutations is unknown. One possibility could be that survival of the clones with high EVI1 expression in combination with a NPM1 or C/EBP-α mutation is impaired.

## Clinical outcome with standard therapy of myeloid malignancies with inv(3)/t(3;3)

Standard front-line treatment of MDS includes DNA de-methylating agents like azacitidine and decitabine. In advanced, high-risk MDS carrying inv(3)/t(3;3) with an increased percent of bone marrow blasts between 5 and 20%, or in overt transformation of MDS to secondary AML (sAML), chemotherapy with cytarabine (Ara-C) and the anthracyclines, idarubicin and daunorubicin is commonly employed. In CML, treatment in the chronic phase generally begins with a tyrosine kinase inhibitor (TKI) such as imatinib, dasatinib or nilotinib. In transformation of CML into blast crisis (CML-BC) carrying inv(3)/t(3;3) or t(3;21), therapy with a second generation tyrosine kinase inhibitors and/or chemotherapy is utilized. EVI1-positive myeloid malignancies have been documented to be relatively refractory to current therapies. There is no statistical difference in the overall 5-year survival rates between MDS and AML with inv(3)/t(3;3), which averages at 3–5%^46,47,51,74^. In a study by the Delwel group, allogenic hematopoietic stem cell transplant following the first clinical remission yielded increased survival odds in AML patients with MLL translocation with EVI1 overexpression^65^. However, greater than 40% of the patients still succumbed to their disease^65^. Overall survival rate of patients with CML who initially respond but later progress on TKI therapy and acquire EVI1 overexpression is half compared to those without EVI1 expression^48^.

### Potential targeted therapies for EVI1-positive myeloid malignancies

To date, following treatment of myeloid malignancies with inv(3)/t(3;3) or EVI1 overexpression with targeted therapies, including DNA hypomethylating drugs, venetoclax or glasdegib, or with FLT3 TKI or IDH1/2 inhibitors, clinical outcome data are unavailable^80^. A targeted agent has yet to be identified and developed that exhibits clinical efficacy specifically against EVI1-overexpressing myeloid malignancies.

### Treatment with ‘epimodifiers’

**Fig. 5:**
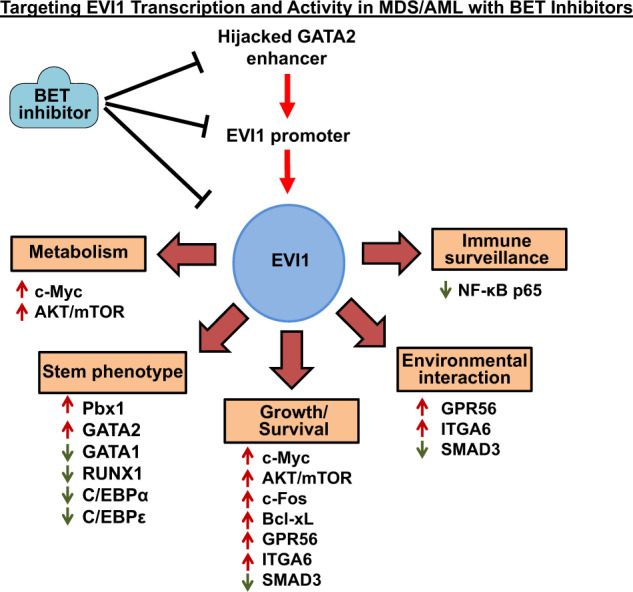
Effects of targeting EVI1 transcription and activity in MDS/AML with BET inhibitors. In AML cells with inv(3)(q21q26) or t(3;3)(q21q26), the hijacked GATA2 enhancer interacts with the EVI1 promoter resulting in high expression of EVI. This leads to aberrant regulation of multiple transcriptional programs including metabolism, stem cell phenotype, growth/survival, environmental interactions, and immune surveillance in AML cells. Treatment with BET inhibitors that evict BET proteins such as BRD4 from the chromatin of the GATA2 enhancer leads to downregulation of EVI1 and its activity in AML cells.

One promising target is the chromatin reader protein BRD4 (bromodomain containing 4), which is involved in transcriptional activation, especially via sustaining the activity of super enhancers, such as those of MYC, CDK4/6 and BCL2/Bcl-xL^81^. By also inhibiting GATA2 super enhancer, treatment with BET (bromodomain and extra-terminal motif) inhibitor could repress *EVI1*, as well as reverse EVI1-dependent transcriptional programs through inactivating enhancers and super enhancers of the key oncogenes (Fig. 5). Preclinical use of BET inhibitor treatment was reported to inhibit growth and induce apoptosis of an EVI1-overexpressing AML cell line^82^. Since several BET inhibitors are already undergoing clinical evaluation, they represent an attractive therapy option for myeloid malignancies with inv(3)/t(3;3) and/or EVI1 overexpression^53^.

Several transcriptional regulators including EVI1 are acetylated by CBP/p300^32^. For example, RUNX1 interacts with EVI1 and is positively regulated by acetylation^32,83,84^. Furthermore, the GATA2 distal enhancer that is relocated and transactivates *EVI1* in inv(3) and t(3;3) chromosomal aberrations contains a p300 binding site that is critical in driving *EVI1* expression^54^. Additionally, the GATA2 enhancer has increased read-through of enhancer RNAs (eRNAs) at the breakpoints that cause its repositioning, and the CBP/p300 inhibitor GNE-049 is reported to particularly inhibit eRNAs^53,85^. Therefore, targeted combination therapy with this HAT inhibitor would simultaneously cause deacetylation of the transcription factors, leading to decreased transcriptional activity, as well as cause the loss of super enhancer function, thereby effectively shutting down EVI1 transcriptional program.

EVI1 can repress transcription through recruitment of DNMT3A or B resulting in de novo methylation^39,86^. EVI1 expression is also associated with hypermethylation of over 200 genes in AML samples^39,86^. Consistent with this, DNA methyltransferase inhibitors have exhibited clinical activity in EVI1-overexpressing AML^87^. However, since monotherapy with DNA hypomethylating agent as a first-line treatment for MDS exhibits only a modest clinical efficacy, combination with other targeted therapies is likely to achieve superior efficacy against EVI1-expressing MDS. Use of EZH2 inhibitor to abrogate dependency of EVI1-expressing MDS or AML with monosomy 7 on the residual normal EZH2 function may exert added efficacy.

### Targeted therapy with BH3-mimetic apoptosis inducers

Recently, co-treatment with venetoclax, a BH3-mimetic inhibitor of Bcl-2 protein, with azacitidine was approved for therapy of AML^88^. EVI1-mediated upregulation of the anti-apoptotic Bcl-xL suggests that BH3-mimetic inhibitor targeting this anti-apoptotic protein could also have therapeutic value, alone or in combinations against EVI1-expressing myeloid malignancies. Recently, the small molecule compound pyrrole-imidazole polyamide, which inhibits the DNA-binding activity of N-terminal ZF domain of EVI1, was shown to induce apoptosis of EVI1-expressing AML cells due to downregulation of the EVI1 target GRP56, which was linked to EVI1-mediated resistance to apoptosis^71^. Several therapies targeting anti-apoptotic proteins are in clinical trials, and these agents could potentially be employed in treatment of EVI1-expressing myeloid malignancies.

### Targeting β-catenin-TCF7L2 activity

In CML with inv(3)/t(3;3), β-catenin/TCF1 (T cell factor 1) signaling was reported to be activated, which positively regulated EVI1^28^. Therefore, along with treatment with BCR-ABL1 targeted TKI inhibition of β-catenin/TCF signaling may be a promising strategy. Additionally, several groups have demonstrated that resistance to BET inhibitors in AML is mediated by the activity of β-catenin/TCF7L2/c-Myc axis, resulting in re-expression of c-Myc despite treatment with BET inhibitor^89^. In this setting also, treatment with a β-catenin/TCF signaling inhibitor, e.g., BC2059 (tegavivint), may not only reverse BET inhibitor resistance but also exhibit synergy with BET inhibitor against AML with inv(3)/t(3;3) and/or EVI1 overexpression^90^.

## Summary and future directions

While great strides have been made, there is still much more to be elucidated regarding EVI1 biology and its contributions to leukemogenesis. EVI1 is a transcriptional regulator that promotes a stem-like expression program in hematopoietic progenitors, crucial to their self-renewal, growth, and repopulating potential. The role of co-expression of alternative EVI1 transcripts in myeloid malignancies remains to be determined. EVI1 regulates transcription through recruitment of epigenetic modifiers. Recent studies have highlighted the dysregulated transcriptome and signaling pathways that underpin the aberrant biology, aggressive phenotype, and refractoriness to standard therapy of EVI1-overexpressing myeloid malignancies (Fig. 5). How commonly the co-occurring genetic alterations and mutations, e.g., monosomy 7 and RAS pathway mutations, and their order of acquisition, contribute to the aggressive phenotype and therapy-refractoriness of EVI1-overexpressing myeloid malignancies has yet to be fully characterized. New probes or alternative methodology need to be developed that will assist in probing the clonal architecture of the co-mutations that occur with EVI1 dysregulation at the single-cell level. Importantly, functional genomic studies need to be conducted to identify specific dependences that can be targeted to achieve superior efficacy against EVI1-overexpressing myeloid malignancies. Large scale screens with or without the presence of a promising therapeutic agent, e.g., by CRISPR technology, could also potentially yield new knowledge for designing effective combination therapies. Analysis of the 3D chromatin architecture of “hijacked enhancers” at EVI1 locus and their response to treatment may also provide insights into effective ways to decrease EVI1 expression, suggest promising treatments, and potential mechanisms of resistance. How dysregulated EVI1 expression in myeloid malignancies creates immune evasion and T cell exhaustion also remains to be fully elucidated. In this exciting era of novel immunotherapies new research avenues and potential strategies have already been illuminated. These are likely to involve harnessing of the innate or adaptive immune mechanisms to overcome immune tolerance or T cell exhaustion in eliminating myeloid malignancies, including those driven by EVI1 dysregulation.
